# DNA methylation and the APP gene: dietary influences in the context of Alzheimer’s disease—a mini review

**DOI:** 10.3389/fnut.2025.1716675

**Published:** 2026-01-16

**Authors:** G. A. Silva-Martínez, J. A. Cervantes-Montelongo, F. E. Tristán-Flores, C. de la Rocha

**Affiliations:** 1 Secretaría de Ciencia, Humanidades, Tecnología e Innovación (SECIHTI), Ciudad de Mexico, Mexico, Mexico; 2 Posgrado de Ingeniería Bioquímica, Departamento de Ingeniería Bioquímica y Ambiental,Tecnológico Nacional de México/IT de Celaya, Celaya, Mexico; 3 Departamento de Ingeniería Bioquímica y Ambiental, Tecnológico Nacional de México/IT de Celaya, Celaya, Mexico; 4 Escuela de Medicina, Universidad de Celaya, Celaya, Guanajuato, Mexico; 5 Departamento de Ciencias Básicas, Tecnológico Nacional de México/IT de Celaya, Celaya, Mexico; 6 Doctorado en Ciencias de la Nutrición Traslacional, Departamento de Alimentación y Nutrición, Centro Universitario de Ciencias de la Salud (CUCS), Universidad de Guadalajara, Guadalajara, Mexico

**Keywords:** Alzheimer’s disease, APP gene, diet, DNA methylation, natural compounds

## Abstract

One of the main pathological features of Alzheimer’s disease (AD) is the accumulation of amyloid *β* (Aβ) peptides, which is encoded by the amyloid precursor protein (APP) gene. The etiology of sporadic AD (non-familial) remains largely unknown, and genetic mutations explain only about 5–10% of cases. Epigenetic alterations, such as DNA methylation, have been proposed as mechanisms contributing to the overproduction of Aβ, which is associated with aggregation. Differences in methylation in the APP gene has been reported in certain brain regions and blood samples from individuals with AD. Dietary components and natural compounds such as fatty acids, methionine, and folate have been associated with differential methylation and may represent potential therapeutic or preventive strategies for AD. This mini-review discusses available evidence on how specific dietary factors and natural compounds may influence DNA methylation of the APP gene revealing promising directions for nutrition-oriented prevention strategies.

## 1 Introduction

Alzheimer’s disease (AD) is the most prevalent neurodegenerative disorder worldwide and the leading cause of dementia, accounting for more than two-thirds of cases (1). It is characterized by progressive cognitive impairment, including memory loss, language disturbances, and behavioral changes. It represents a major and growing challenge for public health systems and caregivers (1, 2). Epidemiological projections estimate that the global prevalence of AD will triple by 2050, exceeding 91 million cases, with annual economic costs surpassing USD 1 trillion (1, 3).

Clinically, AD is classified into early-onset Alzheimer’s disease (EOAD), which represents less than 5% of cases and manifests before the age of 65, and late-onset Alzheimer’s disease (LOAD), which accounts for over 90% of cases (2, 4). EOAD is strongly associated with autosomal dominant mutations in genes encoding the amyloid precursor protein (APP), presenilin 1 (PSEN1), and presenilin 2 (PSEN2), and approximately 35–60% of them have first-degree relatives with dementia (familial AD) (5). In contrast, LOAD arises from complex interactions between genetic susceptibility—most notably the APOE ε4 allele—and diverse environmental and lifestyle factors (2, 4, 6). Both EOAD and LOAD have similar clinical manifestations, and can occur in familial and non familial (sporadic) patients (5).

The neuropathological hallmarks of AD are extracellular amyloid-beta (Aβ) plaques, derived from the amyloidogenic processing of APP, and intracellular neurofibrillary tangles (NFTs) composed of hyperphosphorylated tau protein (3, 7). These lesions drive synaptic dysfunction, neuronal loss, and progressive brain atrophy, leading to the irreversible cognitive and functional decline observed clinically (7, 8). The amyloid cascade hypothesis remains central to the understanding of AD pathogenesis, proposing that aberrant APP processing promotes the accumulation of the neurotoxic Aβ42 isoform, which aggregates into plaques (7, 9). Additional mechanisms—including hypoxia, lipid metabolism disturbances, and glial responses mediated by astrocytes and microglia—further contribute to Aβ deposition and tau pathology, underscoring the multifactorial etiology of AD (8, 10).

Advances in biomarker research, including cerebrospinal fluid (CSF) assays for Aβ42 and tau proteins and neuroimaging techniques such as MRI and PET, have enabled earlier and more accurate diagnosis. Nevertheless, available pharmacological treatments are mainly symptomatic and provide only modest benefits in slowing disease progression (4, 11). Consequently, there is an urgent need to elucidate the molecular and cellular mechanisms underlying AD, particularly the roles of epigenetic regulation and neuroinflammation, in order to identify novel therapeutic targets (6, 12).

Addressing the escalating global impact of AD requires an integrated strategy that combines early detection, management of modifiable risk factors, and the development of disease-modifying therapies directed at the core pathogenic processes of amyloid and tau dysregulation.

## 2 The APP gene and its role in Alzheimer’s disease

The APP gene, located on chromosome 21, produces several isoforms by alternative splicing, with APP695 being the most abundant in neurons (13, 14). The expression of the APP gene is regulated by a variety of mechanisms, including transcription factors that interact with its promoter and enhancer regions, as well as hormones, growth factors, and cytokines that modulate its activity in response to diverse physiological stimuli. These processes have been extensively reviewed elsewhere (15). While APP is often studied for its link to AD, it also serves fundamental physiological functions, such as shaping synaptic connections, supporting neuronal plasticity, and protecting cells from stress (16).

This dual role makes APP both a guardian of neuronal health and, under certain conditions, a contributor to pathology. The outcome of APP activity depends on how the protein is processed. In the non-amyloidogenic pathway, cleavage by *α*-secretase prevents the generation of A*β* and instead produces soluble fragments that promote neuronal survival (17). By contrast, the amyloidogenic pathway, driven by β-secretase (BACE1) and *γ*-secretase, releases Aβ peptides—particularly Aβ42, which readily aggregates into oligomers and plaques (8, 17).

Evidence from genetic studies further supports APP’s role in AD. Rare mutations in APP or in the presenilin genes (PSEN1 and PSEN2) favor A*β*42 production and lead to familial, early-onset AD (18, 19). For example, the “Swedish” double mutation (KM670/671NL) near the β-secretase cleavage site increases total Aβ production, whereas the “Arctic” mutation (E693G) promotes protofibril formation and accelerates plaque deposition (20, 21). These cases are, however, uncommon, representing fewer than 5% of diagnoses (22). Furthermore, most adults with Down syndrome (DS) display neuropathological changes of AD, probably due to extra copies of APP attributed to the trisomy of chromosome 21 (23).

In contrast, sporadic late-onset AD shows no single genetic cause, redirecting attention toward epigenetic mechanisms—DNA methylation (DNAm), histone modifications, and non-coding RNAs—that regulate APP expression in a more flexible and environmentally responsive manner (24, 25).

These epigenetic changes may help explain why APP processing becomes abnormal in sporadic AD even without mutations. Altered DNAm or histone states could link environmental exposures—ranging from diet to toxins, or simply the aging process—to pathological protein accumulation (26–28). Taken together, mutations account for rare hereditary AD, while epigenetic dysregulation may explain the more prevalent sporadic form (29, 30). This dual perspective positions APP as a therapeutic target not only at the genetic level but also through epigenetic programs that regulate its expression.

## 3 DNAm: mechanisms and implications

**Figure 1:**
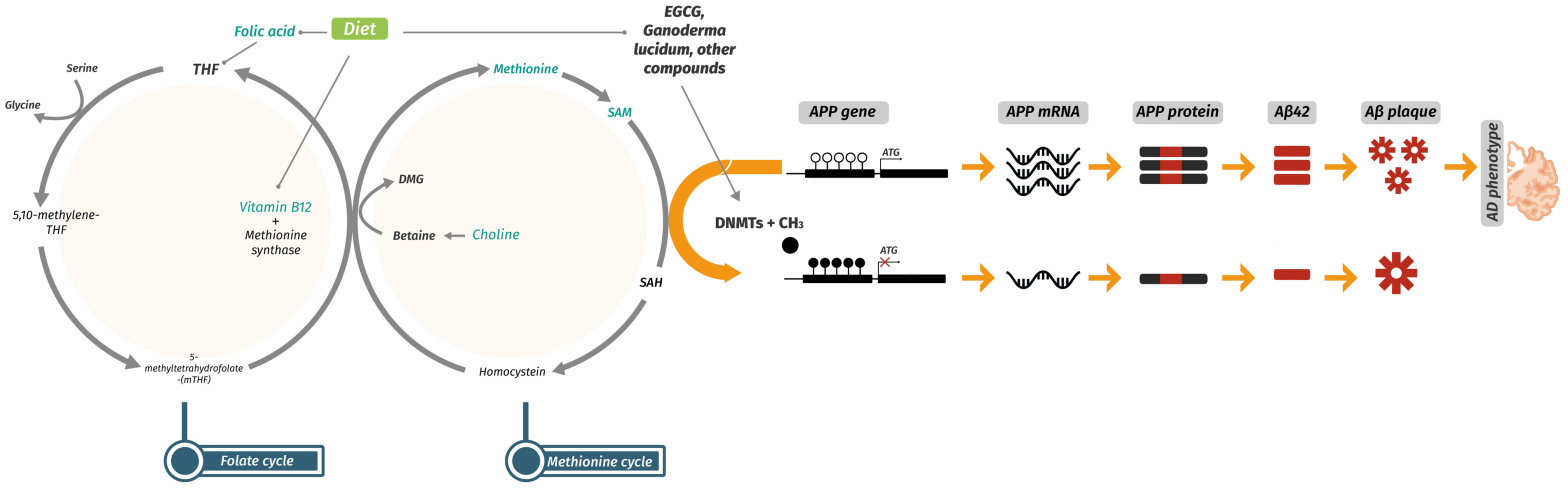
Dietary components and bioactive compounds involved in the one-carbon metabolic cycle and their role in APP promoter DNA methylation. The one-carbon pathway integrates the folate and methionine cycles to produce SAM, the universal methyl donor. Key dietary contributors include folic acid, vitamin B12, choline, betaine, and methionine. In the folate cycle, mTHF is derived from dietary folic acid and, together with vitamin B12, supports the remethylation of homocysteine to methionine via methionine synthase. Methionine is then converted to SAM, which is demethylated to SAH during DNA methylation reactions. DNMTs use SAM as substrate to methylate the APP promoter, resulting in transcriptional repression, reduced protein expression, and probably attenuation of the Alzheimer’s disease–related phenotype. DMG, dimethylglycine; DNMTs, DNA methyltransferases; EGCG, epigallocatechin gallate; mTHF, 5-methyltetrahydrofolate; SAH, *S*-adenosylhomocysteine; SAM, *S*-adenosylmethionine; THF, tetrahydrofolate.

DNAm is one of the most widely studied epigenetic modifications, occurring primarily at the 5-position of cytosine residues within CpG dinucleotides (31). Promoter-associated CpG islands are particularly sensitive to changes in DNAm, making them key sites of transcriptional regulation. DNA methyltransferases (DNMTs) add methyl groups using S-adenosylmethionine (SAM) as the donor, while the TET enzyme family catalyzes active demethylation (32). The functional outcome of DNAm depends strongly on genomic context. Promoter hypermethylation typically represses gene expression by blocking transcription factor binding or recruiting repressive complexes, while gene body methylation is often linked to active transcription (Figure 1). Beyond transcriptional control, methylation also contributes to genomic stability, silences transposable elements, and maintains imprinting patterns (33).

With aging, DNAm undergoes a pattern of global hypomethylation alongside site-specific hypermethylation—a phenomenon termed epigenetic drift (34). This shift may destabilize the genome and alter expression of genes vital for neuronal survival (35). In AD, abnormal methylation has been documented in genes such as APP, BDNF (neurotrophic factor), MAPT (Tau), and PSEN1. For example, hypermethylation of BDNF promoters may weaken neuronal resilience, while hypomethylation of amyloid-related genes, like the APP, may worsen protein accumulation (36, 37).

Overall, DNAm serves as a molecular link between environment, lifestyle, and disease progression. Clinically, this suggests that DNAm profiles could function not only as biomarkers but also as therapeutic targets. This is particularly relevant for the APP gene, whose expression is directly influenced by promoter methylation status, contributing to amyloid pathology in AD.

## 4 Epigenetic regulation of the APP gene

Recent studies indicate that DNAm directly shapes APP expression and may contribute to its misregulation in AD (38, 39). Several studies report hypomethylation of the APP promoter in AD brains, which often correlates with elevated APP expression and greater Aβ burden (36, 40, 41). For example, post-mortem analyses of hippocampal tissue have revealed consistent APP promoter hypomethylation in AD patients, aligning with increased APP transcript levels and plaque density (42). Araki and colleagues demonstrated that reduced availability of methyl donors, such as folate, leads to APP hypomethylation and enhanced amyloidogenic processing in experimental models (43). Similarly, Fuso and colleagues demonstrated in B-vitamin-deficient mice that reduced SAM availability leads to APP and PSEN1 hypomethylation, worsening amyloid pathology—effects that could be rescued with methyl donor supplementation (44). Furthermore, it has been shown in cell lines and murine models of AD that CRISPR/dCAS9-Dnmt3a-directed DNAm of the APP promoter results in a decrease in its expression and accumulation of Aβ42 plaques in the hippocampus and cortex, and improved scores on long-term memory tests (45). Together, these findings point to epigenetic de-repression of APP as a plausible contributor to sporadic AD mainly explained by changes in DNAm in the promoter region (Figure 1).

The LEARn (Latent early-life Associated Regulation) model, proposed by Lahiri et al., supports the above, arguing that environmental factors, such as diet components, and environmental pollutants in early life stages “somatic epitype” induced by maternal behavior, induce latent epigenetic changes that, if maintained or reinforced in adulthood, can result in an AD phenotype, specifically this was evidenced in animal models in the APP (46).

Interestingly, these changes are not uniform across tissues. In vulnerable regions like the hippocampus and cortex, promoter hypomethylation is frequently observed, whereas studies in peripheral blood show mixed results (47, 48). Such discrepancies likely reflect both tissue-specific regulation and methodological differences, but they also underline the potential value of blood-based methylation assays as non-invasive diagnostic tools if standardized methods can be achieved. Histone modifications at the APP locus can either reinforce or counter methylation, altering chromatin accessibility (49). At the same time, non-coding RNAs such as miR-101 and miR-106b modulate APP post-transcriptionally (50, 51). This suggests that APP expression is governed by an interconnected epigenetic network, rather than a single regulatory switch. Overall, because epigenetic states are dynamic and reversible, therapeutic strategies aimed at restoring normal APP regulation may prove especially promising for sporadic AD, in contrast with the fixed mutations that drive familial forms.

## 5 Dietary factors influencing DNAm and modulation of APP methylation in AD

Nutriepigenomics is an emerging field that explores how diet shapes epigenetic modifications and, in turn, influences health and disease (52). In AD, diet is one of the few modifiable factors that may affect methylation of key genes, including APP (53).

### 5.1 One-carbon metabolism nutrients

One of the key modulators of methylation states in diet, is through one-carbon metabolism, which produces SAM the universal methyl donor required for DNAm from its precursor methionine, the by products generated in this cycle are S-adenosylhomocysteine (SAH) and homocysteine (Hcy). Adequate intake of folate, vitamin B12, and related cofactors is necessary to sustain this pathway. Deficiencies disrupt methylation balance and have been linked to hypomethylation of APP and presenilin promoters, changes that aggravate amyloid pathology (54). These diet-driven epigenetic shifts mirror the experimental findings on APP regulation described above, underscoring a mechanistic link between nutrient intake and gene expression.

Folate and B12 deficiencies have been strongly linked to hypomethylation of APP and presenilin promoters in both human and animal models. In APP transgenic mice, folate deficiency worsened amyloid pathology through hypomethylation-dependent mechanisms, while supplementation restored methylation and reduced Aβ accumulation (54, 55). Similarly, SAM supplementation has been shown to reverse DNAm deficits in neuronal cultures and AD models, decreasing APP and PSEN1 expression and lowering amyloid deposition (39, 56), expanding on the experimental findings discussed above.

Interestingly, methionine, while a precursor for SAM, has paradoxical effects. Excess methionine can elevate Hcy levels, which may lead to dysregulated methylation and neurodegeneration. A methionine rich diet in wild type mice, without reaching toxic levels, induced an increment in the levels of both Aβ-42 and Aβ-40, principally in the hippocampus, accompanied by other markers and memory impairment (57). Also, methionine-rich diets increased APP methylation and Aβ expression (58), suggesting context-dependent effects of methyl donors, especially when not balanced by folate and B12 availability (59).

Moreover, maternal dietary choline supplementation in AD mouse models has been shown to improve the disease phenotype, reducing Aβ plaque accumulation and inducing tissue-specific changes in methylation patterns. A recent study evaluated the methylation profiles of AD mice born to supplemented mothers, analyzing hippocampal and cortical tissue across the lifespan. The offspring exhibited differentially methylated genes (DMGs), with hypermethylation observed in the cerebral cortex and hypomethylation in the hippocampus. Notably, most of these patterns were already evident at 3 months of age and persisted up to 12 months. These findings support the use of early-life epigenetic markers as tools for diagnosis or risk detection (54, 55).

### 5.2 Bioactive compounds

Beyond vitamins, dietary polyphenols and other natural compounds also influence methylation. For instance, EGCG from green tea can partially restore methylation of AD-related genes, while resveratrol activates sirtuin signaling with downstream effects on epigenetic regulation through DNAm (60–63). Omega-3 fatty acids have also been linked to improved methylation patterns and better cognitive outcomes, though their direct role in APP methylation remains less clear (64, 65). Although direct effects on APP methylation remain inconsistent, omega-3 may modulate neuroinflammation and indirectly affect epigenetic regulation.

Furthermore, it was observed that mice receiving a methionine-rich diet-induced AD, supplemented with *Dendrobium nobile Lindl.* alkaloids reduced APP protein expression, increased DNMT3a expression, and significantly enhanced APP promoter methylation in the cortex and hippocampus. This was accompanied by decreased levels of Aβ1–40 and Aβ1–42 in both brain tissue and serum, underscoring a potential epigenetic mechanism of action (58). Other compounds that have shown positive outcomes in AD models are *Caliphruria subedentata*, Galantamin (66) and *Ganoderma lucidum* extracts (67) modulating DNAm and preventing neurotoxicity or reducing Aβ1-40-42, respectively.

### 5.3 Dietary patterns

In addition, recent omics-based studies have highlighted the interplay between dietary patterns, DNAm, and cognitive decline. For example, adherence to a Mediterranean diet reduces risk by 11–30% in human cohorts (56) and has been associated with better memory, and less amyloid Aβ42/40 ratios (57) and longitudinally lack of adherence was associated with increased Aβ deposition (58), even though in the above studies no DNAm or epigenetic markers were evaluated, it has been observed that MED adherence in mothers influence offspring methylation (59). Furthermore, the Mediterranean–Dietary Approaches to Stop Hypertension Intervention for Neurodegenerative Delay (MIND) diet—a combination of the Mediterranean and DASH diets—has been proposed as a strategy for dementia prevention and has been associated with a reduced risk of developing AD (60–63), along with lower beta-amyloid burden (64). However, to our knowledge, the relationship between the MIND diet and epigenetic modifications has not yet been explored. To the contrary, animal models fed with Western diets —rich in saturated fats and sugar— generated pathological features in the brain, such as amyloidogenic accumulation of toxic Aβ peptides and tau protein phosphorylation (65) and it has been observed that it exacerbate epigenetic dysregulation (66). Further studies are necessary to elucidate if the MED, MIND or western diet has an impact on the DNAm of AD genes, specifically APP gene.

To illustrate the diversity of findings, Table 1 summarizes representative studies on dietary and natural compounds reported to influence DNAm and Alzheimer’s-related outcomes, including APP regulation. As shown in the table, with the exception of the studies by Frankhouser and Karimi et al. on omega-3 supplementation (67, 68), and the effects of the Mediterranean Diet (57–59) no evidence in humans has been reported.

**Table 1:** Summary of studies reporting the influence of dietary or natural compounds on epigenetic mechanisms in Alzheimer’s disease models.

Nutrient/natural compound/diet pattern	Model/intervention	Reported effects on DNAm	Effects associated with DNAm changes (relevant to APP/Aβ)	Reference
One-carbon metabolism nutrients				
Folate deficiency	APP/PS1 transgenic mice; dietary folate restriction	Promotes global and locus-specific hypomethylation (including amyloid-related loci); reduces expression of some amyloid-associated miRNAs.	↑ APP/PSEN1 expression, ↑ Aβ accumulation, worsened plaque pathology and cognitive deficits.	(83)
Folic acid supplementation	APP/PS1 mice; folic acid administration	Restores methylation at affected loci; normalizes SAM-dependent methylation reactions.	↓ BACE1/PSEN1 mRNA & protein, ↓ Aβ42 deposition, improved biochemical and behavioral endpoints.	(84, 85)
Low folate (cell culture)	Mouse neural stem/progenitor cells; low-folate media	Induces DNA hypomethylation of neuronal differentiation genes and alters gene expression programs.	Impaired neuronal maturation; mechanistic link between low folate and altered neuronal gene regulation (supports *in vivo* findings).	(43)
B-vitamin deficiency → SAM depletion (dietary)	TgCRND8 and other AD mouse models; B-vitamin-deficient diet ± SAM supplementation	B-vitamin deficiency reduces methyl donor availability → DNAm deficits at multiple loci; SAM supplementation restores methylation patterns.	B-vitamin deficiency: ↑ APP-processing enzymes and Aβ; SAM supplementation: ↓ amyloid production, improved spatial memory, reduced PSEN1/BACE1 upregulation.	(44)
Direct SAM supplementation	Transgenic AD mice; SAM dietary treatment	Increases methyl donor pool; rescues DNAm deficits induced by nutrient deficiency and modulates methylation-sensitive genes.	Reduced Aβ and phospho-tau, decreased γ-secretase activity/PSEN1 expression, improved cognition in mice.	(86, 87)
Choline supplementation	Transgenic AD mice; maternal choline administration.	Reduction in the brain Hcy of the offspring of two generations. Hypermethylation in several DMR in cortex and hypomethylation in hippocampus.	Reduced Aβ load and microglia activation, and improved cognitive deficits. No difference in the APP protein expression.	(54, 55)
Methionine	Methionine-rich diet in mice	Increased global methylation and APP promoter methylation	Elevated Aβ-42 and Aβ-40, memory impairment.	(79, 88)
Bioactive compounds				
Omega-3 PUFAs (DHA/EPA)	Human intervention & cohort studies (blood DNAm); animal/intervention studies	Supplementation linked to shifts in peripheral DNAm (global and locus-specific), and to slower changes in DNAm clocks in trials. Effects on brain DNAm less directly proven.	Associated with improved methylation signatures, reduced inflammation, and better cognitive outcomes in some trials — may indirectly modulate APP/Aβ pathways via anti-inflammatory mechanisms.	(67, 68)
EGCG (green tea polyphenol)	*In vitro* neuronal/cancer models and rodent AD models; EGCG treatment	Modulates DNAm and histone marks — effects are context- and gene-dependent (DNMT inhibition) reported in some settings; restoration of DNAm in others.	Changes in expression of APP-processing genes, reduced APP expression in some reports, decreased Aβ aggregation and improved cognition in animal studies (mechanisms multimodal).	(89, 90)
*C. subedentata*, Galantamin	SH-SY5Y cell line	Aβ(1–42) exposition decreased ADNm of APP gene, partially recovered with *C. subedentata* or galant-amine treatments (non-significative). Methylation of BASE and PSEN1 genes was not affected.	Treatment rescues cells from sensibility against Aβ(1–42)-induced neurotoxicity.	(80)
*Dendrobium nobile Lindl.* Alkaloids (DNLA)	C57BL/6 prefrontal cortex and hippocampus	Increased CpG methylation levels of APP and BACE1 genes.	DNLA improved learning and memory dysfunction, alleviated neuron damage. ↓ Aβ1–40 and Aβ1–42 HCY, SAM/SAH, APP, PS1, BACE1, DNMT1. ↑ DNMT3a, DNMT3b.	(79)
*Ganoderma lucidum* Extracts	APP/PS1 and SAMP8 mice (AD models)	Extracts restore CpG methylation in AD mice	↓Aβ1–42 ↑ DNMT3a, DNMT3b Improved behavioral tests.	(91)
Dietary patterns				
Mediterranean diet (MD)	Human cohort studies and meta-analysis	Changes in several CpG sites of offspring	↓Aβ42/40 ↓Aβ deposition	(57–59)
Western diet	Rodents wild type and AD models	Decrease in DNAm globally in frontal cortex, several differentially methylated regions related to neurological function.	↑Aβ1–40 and Aβ1–42, tau, APP	(65, 66)

Taken together, these findings suggest that nutrients influence epigenetic regulation through multiple mechanisms—adjusting the SAM/SAH ratio, modulating DNMT or TET activity, and reducing oxidative or inflammatory stress. Such effects are particularly relevant in the brain, where metabolic balance strongly impacts epigenetic integrity and, ultimately, neuronal survival.

## 6 Discussion

There is increasing evidence that epigenetic dysregulation represents a critical mechanism in the pathogenesis of AD. Among the different epigenetic modifications, DNAm has emerged as particularly relevant. Hypomethylation of the APP gene promoter has been consistently reported in AD brains, leading to elevated APP expression and enhanced Aβ production, a central hallmark of AD pathology (12, 69–71). Similar alterations have been observed in other AD-related genes, including BACE1 and PSEN1, thereby exacerbating amyloidogenic processing and contributing to neuronal dysfunction (72–74).

Dietary factors exert a significant influence on epigenetic regulation. Nutrients such as folate, vitamin B12, and SAM are essential for one-carbon metabolism, which provides the methyl groups required for DNAm. Deficiencies in these nutrients disrupt methylation homeostasis, leading to hyperhomocysteinemia and aberrant expression of genes implicated in Aβ production and neuroinflammatory pathways (74–76). Conversely, dietary interventions—including supplementation with SAM, B vitamins, antioxidants, and omega-3 fatty acids—have shown promise in restoring methylation patterns, attenuating Aβ accumulation, and reducing cognitive decline in preclinical models (72, 77, 78). Furthermore, B-vitamin supplementation has been associated with the prevention of gray matter atrophy in AD-vulnerable regions, underscoring the therapeutic potential of nutritional strategies (69, 77). It has also been shown in human studies that the Mediterranean Diet reduces the risk of developing AD, probably due to changes in the epigenetic marks (56–59). Collectively, these findings highlight the pivotal role of epigenetic mechanisms in AD and suggest that dietary modulation may serve as a preventive or adjunctive therapeutic approach.

However, despite these promising findings, key limitations remain. Notably, no human studies have directly linked dietary exposures to APP-specific DNAm. Nevertheless, recent studies have observed up to 44% homology between differentially methylated regions in AD mice and humans, making mice a good model for analyzing these modifications (55). Furthermore, despite the epidemiological evidence and findings from murine models, to the best of our knowledge, only two experimental studies using bioactive compounds or nutritional components have reported APP methylation as an outcome (79, 80). Consequently, there is a lack of comprehensive analyses examining dietary patterns specifically associated with APP methylation. Both cohort studies and randomized clinical trials are needed to support the development of dietary guidelines.

Furthermore, standardized methods for analyzing methylation patterns and specific regions and tissues are necessary to evaluate the APP gene as a potential biomarker in humans. Gasparroni et al. identified, in postmortem human cortex, a single region (cg08866780) that becomes progressively hypomethylated across Braak stages in both neurons and glial cells (41). Moreover, this same region was found to be hypermethylated in peripheral blood from dementia patients, even prior to diagnosis (81), reinforcing the use of peripheral tissues as non-invasive techniques for biomarker analysis. Further investigation of this locus may be valuable for comparing findings across studies and for evaluating the effectiveness of dietary habits and interventions. Additionally, most murine AD models carry APP mutations; therefore, the effects on methylation within this gene may be influenced by the mutation itself, which also precludes the analysis of APP mRNA transcriptional regulation involving these genomic regions (82). Addressing these gaps will be essential to validate the translational potential of diet-based epigenetic interventions for the prevention or treatment of AD. Also, future research should prioritize the identification of specific epigenetic biomarkers to enable early diagnosis and guide targeted interventions. Personalized nutrition, such as MIND diet, diet rich in one-carbon metabolism nutrients and pharmacological modulation of DNAm represent promising strategies to delay or possibly halt the progression of AD.
